# Can risk factors and risk scores help predict colonization and infection in multidrug-resistant gram-negative bacteria?

**DOI:** 10.1017/ash.2024.455

**Published:** 2024-11-11

**Authors:** Natalia Restrepo-Arbeláez, Juan Carlos García-Betancur, Christian José Pallares, L’Emir Wassim El Ayoubi, Pattarachai Kiratisin, Souha S. Kanj, María Virginia Villegas

**Affiliations:** 1 Grupo de investigación en Resistencia Antimicrobiana y Epidemiología Hospitalaria (RAEH), Universidad El Bosque, Bogotá D.C., Colombia; 2 Division of Infectious Diseases, and Center for Infectious Diseases Research, American University of Beirut Medical Center, Beirut, Lebanon; 3 Clínica Imbanaco Grupo Quirónsalud, Cali, Colombia; 4 Department of Microbiology, Faculty of Medicine Siriraj Hospital, Mahidol University, Bangkok, Thailand

## Abstract

Antimicrobial resistance (AMR) is positioning as one of the most relevant threats to global public health and threatens the effective treatment of an ever-growing number of bacterial infections in various healthcare settings, particularly in acute care and surgical units, as well as in the community. Among multidrug-resistant (MDR) gram-negative bacteria (MDRGNB), *Enterobacterales*, *Pseudomonas aeruginosa,* and *Acinetobacter baumannii* require special attention, since they account for most of the mortality associated with bacterial infections and are often MDR. It is clear that there is an important global variation in antibiotic resistance profiles among MDRGNB species. Extended-spectrum β-lactamase-producing *Enterobacterales*, carbapenem-resistant *Enterobacterales*, DTR-*P. aeruginosa*, and MDR-*A. baumannii* are the focus of this review. Here, we summarize a series of relevant studies on risk factors associated with colonization and infection with these MDRGNB. Likewise, we offer a comparative overview of those studies providing scoring systems to predict the risk of infection with these MDR pathogens, and their pros and cons. Despite the variable accuracy of published risk factors for predicting colonization or infection with MDRGNB, these scores are valuable tools that may help anticipate colonization and infection among those colonized. More importantly, they may help reduce unnecessary use of broad-spectrum antimicrobials and guiding the selection of an optimal treatment.

## Introduction

The prevalence of multidrug-resistant gram-negative bacteria (MDRGNB) is increasing worldwide and is of particular concern in vulnerable populations in acute care settings.^1–5^ Although multiple mechanisms confer β-lactam resistance, β-lactamase production remains the most common mechanism of resistance.^6^ *Enterobacterales*, *Pseudomonas aeruginosa*, and *Acinetobacter baumannii* are identified as some of the most relevant pathogens with high rates of resistance to several classes of antibiotics, limiting treatment options for infections caused by these organisms.^4,7–10^

The rise in MDRGNB poses an increasing problem in acute care, surgery, hospital wards, intensive care units (ICUs), and within the community.^3,8,11–13^ It is important to quantify the burden to have a clear understanding of the epidemiology of MDRGNB, including its regional variability.^4,14–16^ Likewise, it is equally important to understand risk factors for MDRGNB infection to ensure appropriate steps are taken to mitigate their spread and to ensure that appropriate empiric antibiotic therapy is started as early as possible.^17–20^ Not only does delayed appropriate therapy increase the risk of death,^21^ but studies have also shown that it also prolongs hospitalization.^22^ Moreover, prolonged hospitalization places patients at risk for developing subsequent antibiotic-resistant infections,^23^ which can ultimately lead to further antibiotic usage^24^ and exacerbate institutional antimicrobial resistance (AMR) patterns.^25^ Therefore, it is important to investigate whether published current risk factor associations and their related scoring systems accurately identify patients at risk particularly for MDRGNB infections.^26,27^

This review aims to provide an overview of relevant studies on risk factors for colonization and/or infection with MDRGNB, as well as evaluate scoring systems, focusing on those aiming to determine the risk of MDRGNB infection, colonization and/or mortality. We conducted a search for data published in international public databases (PubMed, Scopus, Google Scholar, SciELO—for Latin American and the Caribbean (LAC)—studies and LILACS) dating from January 2014 to June 2023, using as search criteria within the title section and within the abstract section [Title/Abstract] the following terms: “antimicrobial resistance,” “Gram-negative bacteria,” “extended spectrum β-lactamase,” “carbapenem-resistant,” together with [AND/OR] “*Enterobacterales*,*” “Klebsiella pneumoniae*,*” “Escherichia coli*,*”* “*Pseudomonas aeruginosa*,*”* “*Acinetobacter baumannii*,*”* together with [AND/OR] “risk factors,” “risk assessment,” “scoring system,” and “score.” The quality of the studies was not formally evaluated but we have excluded publications not considered as peer reviewed. Duplicates were deleted before screening. Selected documents were reviewed for full-text eligibility and furthermore, we manually selected all those publications reporting a risk score and/or risk assessment to determine the risk of MDRGNB infection and/or mortality. Discrepancies were solved among all authors. During this search, a final number of 81 published studies were finally included in this review and, 23 were used to build the comparative assessment analysis presented particularly in Table 2, as well as in Table 3.

**Table 1.** Single and MDR percentage in isolates of *E. coli, K. pneumoniae*, *P. aeruginosa* and *Acinetobacter* spp. reported by EARS-NET in EU/EEA countries during 2015–2019

	*E. coli* (n = 118,399) (57.1% resistant isolates), combinations used	*K. pneumoniae* (n = 39,025) (36.6% resistant isolates) combinations used	*P. aeruginosa* (n = 18,416) (30.8% resistant isolates) combinations used	*Acinetobacter* spp. (n = 5,696) (53.4% resistant isolates) combinations used
Resistance to one antimicrobial	34.8% Aminopenicillins; fluoroquinolones; other antimicrobial groups	8.0% Fluoroquinolones; third-generation cephalosporins; other antimicrobial groups	13.2% Fluoroquinolones; carbapenems; piperacillin–tazobactam; aminoglycosides; ceftazidime	4.8% Fluoroquinolones; aminoglycosides; carbapenems
Resistance to two antimicrobials	10.5% Aminopenicillins + fluoroquinolones; aminopenicillins + third-generation cephalosporins; aminopenicillins + aminoglycosides; other antimicrobial group combinations	8.1% Third-generation cephalosporins + fluoroquinolones; third-generation cephalosporins + aminoglycosides; fluoroquinolones + aminoglycosides; other antimicrobial group combinations	7.6% Piperacillin–tazobactam + ceftazidime; fluoroquinolones + carbapenems; other antimicrobial group combinations	5.0% Fluoroquinolones + carbapenems; fluoroquinolones + aminoglycosides; aminoglycosides + carbapenems
Resistance to three antimicrobials	7.3% Aminopenicillins + third-generation cephalosporins + fluoroquinolones; aminopenicillins + fluoroquinolones + aminoglycosides; other antimicrobial group combinations	15.6% Third-generation cephalosporins + fluoroquinolones + aminoglycosides; third-generation cephalosporins + fluoroquinolones + carbapenems; other antimicrobial group combinations	3.9% Piperacillin–tazobactam + ceftazidime + carbapenems; other antimicrobial group combinations	43.6% Fluoroquinolones + aminoglycosides + carbapenems
Resistance to four antimicrobials	4.5% Aminopenicillins + third-generation cephalosporins + fluoroquinolones + aminoglycosides; other antimicrobial group combinations	4.9% Third-generation cephalosporins + fluoroquinolones + aminoglycosides + carbapenems	2.8% Piperacillin–tazobactam + fluoroquinolones + ceftazidime + carbapenems; other antimicrobial group combinations	–Data not available
Resistance to five antimicrobials	<0.1% Aminopenicillins + third-generation cephalosporins + fluoroquinolones + aminoglycosides + carbapenems	–Data not available	3.4% Piperacillin–tazobactam + fluoroquinolones + ceftazidime + aminoglycosides + carbapenems	–Data not available

Data source: EARS-Net, European Antimicrobial Resistance Surveillance Network; EU/EEA, European Union/European Economic Area.^45^

**Table 2.** Comparative analysis of risks factors for MDR-GNB infections or colonization on evaluation studies

Ref.	Type of study	Population	Defined case or event	Risk factor^1^ (for infection or for colonization)											
				Previous use of antibiotics	History of previous hospitalization	Invasive procedures or surgeries	Urinary catheterization	Referral from another hospital	Previous colonization	Immunosuppression	Advanced age	Diabetes mellitus	Previous history of infection	Type of infection	Other
^89^	Case-control	110 cases: 338 controls	ESBL Infection: *E. coli and K. pneumoniae*	3.8^24^ (2.1–6.9)	3.2^12^ (1.6–6.5)		5.9^4^ (3.1–11.6)	4.3 (2.1–8.8)							
				3.5^24^ (1.9–6.4)	2.6^12^ (1.3–5.4)		6.9^4^ (3.6–13.4)	5.3 (2.7–10.7)		2.3^8^ (1.1–4.8)					
^90^	Cohort	184 participants (76 events)	Colonization: *P. aeruginosa* carbapenem-resistant	9.9^25^ (1.9–51.4) 2.5^3,^^26^ (1.1–5.5)								3.9 (1.5–10.2)			
^84^	Cohort	11502 participants with infection (2324 events carbapenem-resistant and 1033 BRE^2^)	Infection: *P. aeruginosa* susceptible, carbapenem-resistant and EBR	4.2^4,^^5,^^26^ (3.3–5.3) 2.3^4,^^6,^^26^ (1.7–2.9)									8.8^5^ (6.7–11.5) 5.0^6^ (3.9–6.5)		
^91^	Case-control	192 cases432 controls	Infection by MDRGNB^7^							1.9^9^ (1.1–3.2)				23.5^10^ (7.0–79.1)	
^68^	Cohort	334 participants (UCI)	Infection by CRE^11^		6.6^13^ (1.3–34.3)	3.7^14^ (1.2–11.1)			10.8^15^ (2.8–41.9)						
^92^	Case-control	91 cases: 1376 controls	Colonization MDRGNB^7^		1.01^12^ (1.00–1.03)	2.8^16^ (1.3–5.9) 2.0^14^ (0.9–4.0)			5.3^7^ (1.5–16.6)						6.5 (2.2–19.2)
^54^	Case-control	143 cases: 572 controls	InfectionCR-KPE^18^			1.9^16^ (1.2–3.0)			3.4^18^ (2.5–4.4)	3.1^8^ (1.8–5.3)					1.6^29^ (1.1–2.6)
^52^	Case-control	113 cases: 339 controls	InfectionESBL-EKP^19^	3.7^24^ (2.0–6.9)	5.7^12^ (2.9–11.0)		3.5 (2.0–6.9)	5.6 (1.6–19.1)			3.2^20^ (1.8–5.7)				
^82^	Cohort	1141 participants (65 events)	Infection ESBL: *E. coli, K. pneumoniae, P. mirabilis, K. oxytoca*	15.3 (7.7–61.4)	3.7 (1.6–9.00) 10.0^21^ (5.0–20.1) 27.8^22^ (12.6–61.3)	12.3^14^ (5.6–27.2)	3.4 (1.1–10.2)					2.1 (1.1–4.1)			
^83^	Case-control	443 cases: 367 controls	Infection by *E. coli* ESBL	2.1 (1.4–3.2) 12.9^32^ (4.4–37.8)							1.5^27^ (1.0–2.2)		7.9^31^ (2.2–27.7)	2.3^28^ (1.5–3.5)	1.9^30^ (1.1–3.2)
^49^	Cohort	336 cases: 59 controls	InfectionESBL-EKP^19^	3.2 (1.7–6.2)							2.4^34^ (1.2–4.9)			5.4^10,36^ (2.9–10.2)	6.9^35^ (1.6–31.0) 3.1^33^(1.2–8.0)

^1^ Risk factors are expressed in the association measure Odds Ratio (OR) with 95% confidence intervals, ^2^ BRE: resistant to carbapenems, ceftazidime and piperacillin/tazobactam, ^3^ within the last 3 months, ^4^ within the last 30 days, ^5^ *P. aeruginosa* carbapenem-resistant infection, ^6^ *P. aeruginosa* BRE *infection,* ^7^ gram-negative bacilli resistant to 3 different antibiotic families, ^8^ immunosuppressive therapy with steroids, tacrolimus, sirolimus, cyclosporine, mycophenolate, antithymocyte globulin, or chemotherapy (with alkylating agents) within the past 3 months, ^9^ Steroids within the past 3 months, ^10^ urinary tract infection, ^11^ carbapenem-resistant *Enterobacteriaceae*, ^12^ within the last year, ^13^ within the last 6 months, ^14^ Esophagus-gastro-duodenoscopy or colonoscopy within the past 6 months, ^15^ previous colonization by CRE, ^16^ abdominal surgery a year or less ago, ^17^ hospital stay longer than 5 days, ^18^ *carbapenem*-resistant *Klebsiella pneumoniae*, ^19^ ESBL *E.coli, K. pneumoniae* and *P.mirabilis*, ^20^ age ≥ 70 years, ^21^ emergency room consult within the last 4 weeks, ^22^ nursing home resident, ^24^ β-lactams or quinolones within the past 3 months, ^25^ aminoglycosides in the last 3 months, ^26^ carbapenems,^27^ age ≥ 55 years, ^28^ hospital-acquired infection, ^29^ gospitalization in ICU,^30^ prolonged hospitalization, ^31^ previous infection with *E.coli* ESBL,^32^ 3^rd^ or 4^th^ generation cephalosporins, ^33^ liver cirrhosis, ^34^ age > 60 years, ^35^ chronic obstructive pulmonary disease, ^36^ intra-abdominal infection.

**Table 3.** Performance of risk scores predicting infection, colonization, or mortality by MDR bacteria

Score reference	Sensitivity	Specificity	Negative predictive value
Giannella, *et al.* 2014^54^ GRS	93%	42%	96%
Cano *et al.* 2018^27^ GRS & ICS	93%	87%	94%
Tumbarello *et al.* 2011^52^	93%	62%	97%
Johnson *et al.* 2013^85^	87%	69%	94%
Kengkla *et al.* 2016^83^	*74%*	66%	68%
Lee *et al*. 2017^82^	85%	93%	99%
Henderson *et al.* 2020 PBS^*^ and qPitt^**^^86^	95%	65%	n.d.^***^
Henderson *et al.* 2020 PBS and qPitt^86^	93%	60%	n.d.
Teysseyre *et al.* 2019^87^ CarbaSCORE	96%	91%	n.d.

* PBS score.

** qPitt score.^86^

*** n.d. = no data.

## Global burden of bacterial AMR

One of the most comprehensive studies evaluating AMR burden was published in 2022.^28^ The study assessed estimated deaths and disability-adjusted life-years (DALYs) directly attributed to bacterial AMR. It examined the deaths and DALYS associated with 23 different pathogens and multiple pathogen–drug combinations in 204 countries in 2019.^28^ The researchers used systematic literature reviews, hospital systems, surveillance systems, and other sources to obtain a wide range of data.^28^ Furthermore, they employed predictive statistical modeling to estimate AMR burden, even in locations lacking specific data.^28^ In summary, three infectious syndromes were associated with the highest AMR burden in 2019: *(i)* lower respiratory infections, *(ii)* bloodstream infections, and *(iii)* intra-abdominal infections.^28^ These three syndromes accounted for 79% of deaths attributed to AMR in 2019. Notably, six pathogens were responsible for more than 250,000 deaths associated with AMR; those pathogens were: *E. coli*, *S. aureus*, *K. pneumoniae, Streptococcus pneumoniae, A. baumannii,* and *P. aeruginosa*. Although the order of attributable mortality of the pathogens varied depending on the world’s region, together, these six pathogens were responsible for a calculated 929,000 deaths attributed to AMR, and 3.57 million deaths associated with AMR globally in 2019.^28^

As the prevalence of MDR organisms is constantly increasing, along with its associated mortality, identifying risk factors for developing an MDRGNB infection could greatly affect patient care and infection management.^20,29^

The increase in AMR is a global concern; the World Health Organization’s (WHO) in its most recent Global Antimicrobial Resistance and Use Surveillance System (GLASS) report from 2022, involving surveillance data from 109 countries collected during 2017–2020, revealed multiple concerning findings including, increased rates of MDR *E. coli* from blood isolates by more than 15% in 2020 when compared to the rates of 2017, particularly for meropenem (0.5% [in 2017]–0.9% [in 2020]) and third generation cephalosporins (20.2% [in 2017]–24.0 [in 2020]). Similarly, they reported high levels of resistance in *K. pneumoniae* to third-generation cephalosporins (59%–64.7%) and to fourth-generation cephalosporins (57.4%), with isolates often showing multidrug resistance. Also, a high percentage of resistance to carbapenems in *Acinetobacter* spp. (> 50%), 73.1% (in 2017) and 72.9% (in 2020).^30^

### Overview of AMR in GNB: regional dissemination

Multiple antibiotic-resistant organisms have spread worldwide; particularly extended-spectrum β-lactamase (ESBL)-producing *E. coli*. in India and Latin America, this group represents up to 40% of the worldwide burden of clinical isolates of ESBL-producing *E. coli*.^31,32^ CR-*K. pneumoniae* is common (6%–28%) in China, the US, and Latin America. New Delhi metallo-β-lactamase (NDM)-producing *Enterobacterales* have been increasingly observed over the past 15 years in South Asia, China, the USA, and Europe.^31,33–35^ Variants of several β-lactamase genes, particularly ESBLs, such as *bla*_CTX-M_, *bla*_TEM_, *bla*_SHV_, *bla*_PER_, *bla*_VEB_ and *bla*_TLA_ variants, are reported worldwide, among which *bla*_CTX-M_ group is the most prevalent group.^36^ Carbapenem-hydrolyzing enzymes such as *K. pneumoniae* carbapenemases (KPC), NDM-1, the imipenemases (IMP), Verona integron-encoded metallo-β-lactamases (VIM) and OXA (oxacillinase)-48 have become widely disseminated. However, there are important differences in the prevalence of these enzymes across countries and particular geographical regions.^37,38^ The successful global dissemination of these carbapenemases is primarily attributed to the presence of their genes on mobile genetic elements, most commonly plasmids. These elements often co-harbor resistance to multiple classes of antibiotics, leading to the emergence of pan-drug-resistant organisms.^39,40^

The regional and national variations, whether in terms of ESBLs or carbapenemases, are mainly due to variations in population density, hygiene and integrity of the country’s infrastructure, use of antibiotics, and infection control practices.^41^ As for its international spread, it was attributed largely to international travel, including globalized medical tourism.^42^

Globalization, along with the increase in antibiotic use worldwide, has significantly affected the evolution and spread of antibiotic-resistance genes over the past 25 years. Studies have shown that 8% of *E. coli* isolates in the US produce ESBL, while in Latin America and South Asia the percentage may reach 32% and 33%, respectively.^43,44^ In 2019, more than half of *E. coli* isolates reported to EARS-Net and more than a third of *K. pneumoniae* isolates were resistant to at least one antimicrobial group of interest, and combined resistance to multiple antimicrobial groups was common.^45^ While carbapenem resistance remained rare in *E. coli*, in contrast, several countries reported carbapenem resistance percentages above 10% for *K. pneumoniae*. In this report, carbapenem resistance was also common in *P. aeruginosa* and *Acinetobacter spp*.^45^ A summary of this international report, covering more than 30 countries, is given in Table 1.

## Risk factors and risk assessment scores for colonization and infection by MDRGNB

Infections caused by MDRGNB mainly involve carbapenem-resistant (CR) and ESBL-producing pathogens. Most of these infections are healthcare-acquired; however, some originate in the community.^46,47^ Multiple publications have addressed risk assessment for ESBL-producing and CR- related infection and colonization. Table 2 describes and compares studies that identified risk factors associated with these conditions and their corresponding odds ratio.

It is currently uncertain whether it is necessary to conduct active fecal screening for ESBLs at hospital admission.^48^ The prevalence of ESBL-producing *Enterobacterales* (ESBL-PE) infections among carriers at ICU admission was reported with a rate of 15% in a recent 7-year study.^49^ From this study, a clinical risk score designed to predict ESBL-PE infection was derived from five independent risk factors associated with ESBL-PE infection in carriers (ie, age >60 years, cirrhosis, broad-spectrum antibiotic treatment within the previous 3 months, urinary or intra-abdominal infection, and absence of chronic pulmonary disease). Based on these variables, an ESBL risk score was created to guide empirical carbapenem therapy against ESBL-producing *Enterobacterales* infections, scores of 4–5, and 6–7 had a prevalence of 26%, and 49%, respectively.^49^ Based on a systematic review and meta-analysis, ESBL colonizers were more likely to have a history of recent antibiotic treatments or healthcare facility utilization.^50^ However, predicting colonization is not easy; many risk factors are nonspecific and are also common to all MDRGNB.^51^ Nevertheless, some scores of risk stratification reliably identify colonization by resistant bacteria. An example is a scoring system devised by Tumbarello *et al.*, which is used to identify ESBL colonization in patients with GNB infections (*E. coli*, *Klebsiella* spp. or *Proteus mirabilis*) at hospital admission.^52^

Conversely, CRE infections have become an urgent public health threat with higher morbidity and mortality, as well as limited antibiotic treatments available.^53^ Although the CDC does recommend surveillance for CR gram-negative pathogens for infection control purposes, some studies also have shown that the detection of colonization by a CR organism (CRO) could guide empirical therapy in certain populations such as hematology patients or kidney transplant recipients.^48^ The primary risk factors for developing CRE infections include longer hospital stays, ICU admission, invasive procedures, recent surgery, recent immunosuppression/immunodeficiency, severe underlying comorbidities, prior antibiotic exposure, and colonization/carriage with CRE.^27,54^ Additional studies indicate that ICU admission, transfer between wards, prolonged hospital stay and sharing a room with known carriers are predominant factors related to CRE colonization.^55,56^ There is also evidence showing high rates of CRE infections up to 45% in critically ill individuals among patients who were previously colonized but asymptomatic.^57,58^ A systematic review of 92 studies found that the most important risk factors for CR-GNB include previous antibiotic use (especially carbapenems), previous colonization, mechanical ventilation, previous ICU stay, dialysis, catheter use, length of hospital stay, comorbidities and, an increase in Acute Physiology and Chronic Health Evaluation (APACHE) II score.^19^ To better predict the development of infections, risk factors have been evaluated and scored for carriers of these CR bacteria.

The Giannella risk score (GRS) has been used to identify the risk of KPC-producing *K. pneumoniae* bloodstream infection (BSI) among rectal carriers.^54^ Notably, multi-site colonization was considered the most reliable predictor.^54^ Further cohort studies have measured the utility of the GRS and determined a cutoff point (of 7) to discriminate between low and high risk of all KPC-producing *K. pneumoniae* infection.^27^

Lastly, the acquisition of MDR *P. aeruginosa* is significantly related to ICU admission but is also related to other factors including prior use of broad-spectrum (cephalosporins, aminoglycosides, quinolones, carbapenems) high invasive-device scores and prior hospital stays, advanced age, and human immunodeficiency virus infection.^9,52,59–61^ In the case of MDR *A. baumannii* infections, prior colonization plays an important role as a risk factor; as well as, previous antibiotic use, prolonged or prior hospitalization, previous ICU stay, or invasive procedures and repeated skin grafts.^62–65^

Based on previous studies and the fact that previous colonization with a MDRGN can lead to an infection, rectal surveillance in patients hospitalized in critical care areas in hospitals with high prevalence of MDRGN is suggested, as this practice can help guide treatment for critically ill patients.^66–68^

## Risk factors and risk scores for mortality in MDRGNB

Data from the WHO confirms that the risk of death attributable to infection by antibiotic-resistant microorganisms is twice that of non-resistant bacteria.^69^ Falagas *et al.* published a meta-analysis of nine studies on mortality following CRE infections in 2012, reporting that 26%–44% of deaths in seven studies were attributable to carbapenem resistance.^69^ A recent multinational prospective cohort study that looked at the effect of carbapenem resistance on outcomes of BSI caused by *Enterobacterales* in low- and middle-income countries (LMICs) showed that carbapenem resistance was associated with increased length of hospital stay and mortality.^70^ In Latin American countries, BSI by CRE has been shown to increase in-hospital mortality four times, compared to non-CRE infection (OR = 4.0 CI 95% (1.7–9.5)).^71^ However, an analysis of 24 studies on infections caused by MDRGNB in ICUs published in 2016, did not confirm a direct association between infections due to MDRGNB and mortality in ICU patients.^72^

Many factors confound the identification of main risk predictors for mortality in MDRGNB-associated infections, including variability in patient populations, late onset of appropriate initial antibiotic therapy, lack of appropriate antibiotic therapy on site in everyday practice, heterogenicity in the outcomes of each study, and the lack of availability of a quick and specific diagnostic test to identify MDR bacteria.^3^ Despite inconsistencies in studies defining risk factors, here we point out studies with common risk factors reported to be associated with higher risk for mortality in MDRGNB.

Patolia *et al.* conducted a retrospective observational cohort study and used data collected over a 13-month period from the electronic health records of patients with gram-negative bacteremia at a single university medical center; 177 patients were included in the analysis, 46 of which (26%) had MDRGNB bacteremia with a mortality rate of 34.8%, compared to 13.7% in the non-MDR-gram-negative bacteremia group (*P* = 0.002). In particular, inappropriate empiric antibiotics (OR: 7.59, 95% CI: 1.68–34.34), urinary catheter as a source of infection (OR: 5.68, 95% CI: 1.37–23.5), intra-abdominal source of infection (OR: 3.66, 95% CI: 1.14–11.73), end-stage liver disease (OR: 3.64, 95% CI: 1.07–12.3) and solid organ malignancy (OR: 3.64, 95% CI: 1.25–10.56) were significant independent risk factors for mortality in patients with MDRGNB. Additional risk factors were identified in the multivariate analysis as significant for the development of a MDRGNB infection: diabetes mellitus (OR: 2.8, 95% CI: 1.00–4.88), previous antibiotic use (OR: 2.93, 95% CI: 1.25–6.87), and urinary catheter as a source of infection (OR: 5.96, 95% CI: 1.78–19.94).^20^

For *A. baumannii*, significant mortality has been reported with CR-*Acinetobacter baumannii* (CRAB) infection although limited data on contributing microbiological factors have been published. In a retrospective study of 164 patients, Hyo-Ju Son *et al.* found that 90 (55%) of the 164 patients died within 30 days.^73^ In the multivariate analysis of study findings, independent risk factors for mortality were a non-eradicated focus, septic shock, and inappropriate antimicrobial therapy.^73^ A recent meta-analysis evaluating the predictors of mortality in patients infected with CRAB identified that the most relevant risk factors for mortality were inappropriate empirical antimicrobial treatment (OR, 5.04; 95%), septic shock (OR, 5.65; 95), chronic liver (OR, 2.36; 95%), and chronic renal disease (OR, 2.02; 95%).^74^

Infection with MDR *P. aeruginosa* is also associated with high mortality. In a retrospective study of patients diagnosed with *P. aeruginosa* BSIs in two Italian university hospitals, Tumbarello *et al*.^75^ compared risk factors for isolation of MDR or non-MDR *P. aeruginosa* in blood cultures. Presentation with septic shock, infection due to MDR *P. aeruginosa* and inadequate initial antimicrobial therapy were the variables independently associated with 21-day mortality.^75^

For CP-CRE, Tamma *et al.* conducted an observational study that compared 14-day mortality in patients with CP-CRE and non-CP-CRE bacteremia. After adjusting for severity of illness on day 1 of bacteremia, underlying medical conditions, and differences in the antibiotic treatment administered, the odds of dying within 14 days was more than four times greater for CP-CRE compared with non-CP-CRE bacteremia patients (aOR, 4.92; 95% CI: 1.01–24.81).^76^

The INCREMENT CPE score was developed, and subsequently validated, to assess mortality risk – and subsequently guide the initiation of empiric therapy – using objective clinical criteria in CPE-colonized patients, including KPC-producing *K. pneumoniae*. The underlying logistic regression model identified the following variables in BSIs and assigned a point-based weighting, to which a threshold was applied.^27,77^ INCREMENT-CPE has been externally validated in further studies.^78,79^

Finally, a meta-analysis published by Vardakas *et al.* included 30 studies, 25 of which were retrospective, nine provided data on predictors of mortality for MDRGNB infections only, and 21 provided data for MDRGN versus non-MDRGNB infections.^80^ Within the studies, *Acinetobacter* spp., *P. aeruginosa* and *Enterobacterales* were the most studied bacteria. There was significant diversity among studies regarding evaluated predictors of mortality. Nevertheless, the most reported independent predictors of mortality were infection severity and underlying diseases, followed by multidrug resistance, inappropriate treatment and increasing age. In the studies that included only patients with MDRGNB infections, cancer (RR: 1.65, 95% CI: 1.13–2.39) and prior or current ICU stay (RR: 1.27, 95% CI: 1.02–1.56) were the risk factors associated with mortality.^80^

## Author’s opinion: advantages and disadvantages of risk scores

There are multiple advantages of the scoring systems; they are simple to calculate and are based on readily available data at hospital admission.^54,75,81,82^ Such scores can shorten the time to initiate appropriate empiric therapy, help optimize therapy and avoid inappropriate and potentially toxic therapies.^27,54,83,84^ Such scoring systems also help de-escalate therapies, avoiding toxicity and costs associated with extended antibiotic use.^52,54^ In studies evaluating these scoring systems, those assessing MDRGNB risk performed well, providing significant and accurate predictions of infection or mortality with good powers of prediction.^27,52,54,83^ Table 3 summarizes the different studies evaluated for this review and compare the different risk scores for the prediction of infection or mortality by resistant bacteria. We found that the sensitivity of scores ranges between 74%–96%, the specificity from 42%–91%, and the negative predictive values from 68%–99%.^27,52,54,82,83,85^

Despite the advantages of using risk scores, we found that one of the major disadvantages with MDRGNB risk scoring systems is that multiple methods have not been externally validated in wider populations and may not be generalizable to patient cohorts in different regions.^27,54,81–83,86,87^ In addition, it was not clear which sub-populations would benefit most from this risk assessment, as some studies evaluated specific populations^21,43,73,76^ or specific sites of infection.^43,70,76^ The utility of a scoring system depends on the intended application and its ultimate value. Moreover, many of the studies assessing these scores have been retrospective, and do not consider the different methods and patient populations involved.^82,86,88^ It is also worth noting that the quality of data and medical practice may not be consistent across all sites.^82,86,88^ Moreover, scoring systems are unable to determine the duration of carrier status prior to the development of an infection.^54^ In some scoring systems, the cutoff value for the score that represents a risk is critical and can change the indicated outcome, and more data is needed to determine the reliability of risk prediction. Due to the difference in rates of resistance at various locations, some scoring systems may also require local adaptation of the test parameters or changes in cutoff values.^85^ Another limitation is that most risk scoring systems are intended for use with specific pathogens/resistance mechanisms rather than multiple different mechanisms of resistance of GNB, and this may restrict their usefulness and widespread use, or dictate that multiple different scores may need to be used.^81,85–87^ Nonetheless, examples like the INCREMENT-ESBL scoring system, which has been evaluated at multiple different locations internationally, has more reliable evidence-based data to support its use. Despite these current drawbacks, risk scoring systems show promise and have the potential to expedite appropriate treatment and substantially improve outcomes in serious MDRGNB infections.

## Conclusions

This review underscores the concerning prevalence of antibiotic resistance in MDRGNB, specifically focusing on the clinically relevant *Enterobacterales*, *P. aeruginosa*, and *A. baumannii*. The escalating global presence of these bacteria emphasizes the critical need to identify colonization and infection risk factors which are significantly linked to mortality. Selecting multiple publications which reported risk factors with association measures and compared the different OR values that might aid when assessing risk factors to predict colonization and/or infection with MDR bacteria may help the selection of empiric treatment in a more appropriate way. Although the accuracy of published risk factor scores and assessments varies, it is imperative to consider these factors and utilize available tools judiciously. We found that most risk scores have a high sensitivity, while most of them are not specific. Nonetheless, we believe that used in a judicious and individualized way in each clinical situation, these tools can aid healthcare workers in expediting appropriate empiric therapy, guiding the selection of optimal treatment, applying necessary infection control and prevention measures and thereby improve the overall clinical outcomes for patients. In the future, machine learning, as well as AI, could revolutionize the prediction of colonization and infection risk. By combining patient-specific risk factors with dynamic, machine-learning-driven approaches, these advanced models have the potential to significantly enhance predictive accuracy. Unlike traditional methods, machine learning models and AI can integrate vast amounts of data, including clinical, microbiological, and demographic information, in real time. This allows for the generation of personalized risk assessments tailored to each patient’s unique profile, enabling earlier and more targeted interventions.

However, it is important to note that while these risk assessment tools can be valuable in initiating empirical therapy for at-risk patients, they should not supersede clinical judgment or the availability of clinical data to prevent the overuse of broad-spectrum antibiotics.
